# Per- and Polyfluoroalkyl Substances in Cosmetics and Personal Care Products: State of the Art in Human Exposure

**DOI:** 10.3390/toxics14100930

**Published:** 2026-10-20

**Authors:** Annalisa Abballe, Isotta Cursi, Valentina Marra, Noemi Milia, Silvia Valentini, Anna Maria Ingelido

**Affiliations:** Unit of Human Exposure to Environmental Contaminants, Department of Environment and Health, Istituto Superiore di Sanità, Viale Regina Elena 299, 00161 Rome, Italyisotta.cursi@iss.it (I.C.); annamaria.ingelido@iss.it (A.M.I.)

**Keywords:** PFAS, human exposure, cosmetics, skin care products, personal care products (PCPs), dermal exposure, dermal absorption

## Abstract

Per- and polyfluoroalkyl substances (PFASs) are a large group of synthetic chemicals widely used since the 1950s due to useful properties such as water resistance and durability. Their stability makes them persistent environmental contaminants, often called “forever chemicals.” Thousands of PFASs exist and many of them have been linked to adverse health effects. The increase in knowledge about PFASs has led to regulations on their production and use, at least to reduce their presence in the main sources of human exposure, like food and drinking water. Other sources and exposure routes have been less explored and deserve more attention for proper risk management. Indeed, many PFASs continue to be used in numerous categories of applications. Among those that have not yet received much attention in terms of consumer awareness are personal care products (PCPs) and cosmetics. Cosmetics and PCPs represent an underexplored but potentially important exposure pathway, especially through dermal contact. Studies show that PFASs are present in various products such as foundations, mascaras, sunscreens, and lotions, sometimes even when not listed as ingredients. Biomonitoring studies also indicate associations between cosmetic use and PFAS levels in human tissues. Research remains limited and inconsistent due to the diversity of PFASs, lack of standardized methods, and scarce toxicological data. This review aims to highlight our level of knowledge on the subject, as well as data gaps, to provide guidance for future studies.

## 1. Introduction

Per- and polyfluoroalkyl substances (PFASs) are a large group of synthetic compounds used in many industrial, commercial and consumer applications since the 1950s. They are widely used in carpets, heat-resistant non-stick cooking surfaces, wall paint, firefighting foam, food packaging, furniture, and cosmetics and personal care products (PCPs) [1,2,3,4,5,6]. These substances are characterized by carbonaceous chains (linear or branched) in which some or all of the hydrogen atoms are replaced by fluorine atoms. The strength of the C-F bond ensures the stability of these molecules that are often referred to as “forever chemicals” due to the chemical stability, making them persistent environmental contaminants. Variations in chain length, type of functional group and molecular structural characteristics give these compounds different physicochemical and toxicological properties. The exact number of PFASs in global commerce is unclear due to varying definitions and methodologies. In 2018, the OECD identified over 4700 PFASs by CAS numbers [7], some of which have been more widely used and studied than others.

PFASs include perfluoroalkyl carboxylic acids (PFCAs) and perfluoroalkyl sulfonic acids (PFSAs); perfluoroalkyl sulfonic acids with six or more C atoms and perfluoroalkyl carboxylic acids with seven or additional C atoms are deemed long-chain PFASs and are known as legacy PFASs [8]. Perfluorooctane sulfonate (PFOS) and perfluorooctanoic acid (PFOA) are two of the most widely studied long-chain PFASs that have been detected in various environmental compartments [9]. Having been recognized as persistent substances, PFOS, its salts and perfluorooctane sulfonyl fluoride (POSF) were listed in 2009 under Annex B of the Stockholm Convention on Persistent Organic Pollutants (POPs) [10]. In 2019 and then in 2022, PFOA and PFHxS together with their salts and related compounds were also included in Annex A [11,12]. Finally, in November 2023, the International Agency for Research on Cancer (IARC) classified PFOA as “carcinogenic to humans (Group 1)” and PFOS as “possibly carcinogenic to humans (Group 2B)” [13]. In recent years, due to the phase-out of certain PFASs, new compounds have been produced as alternative substances.

These emerging PFASs, such as short-chain PFASs (usually referred to as C4 to C6) and ultra-short chain PFASs (usually referred to as C1 to C3), and HFPO-DA/GenX, 6:2 Cl-PFAES, and PFECHS, are a cause for great concern because, once released into the environment, they exhibit similar persistence, mobility, and potential toxicity [14,15].

Due to their widespread production and use, as well as their ability to move, persist and bioaccumulate in the environment, PFASs can be transported over very long distances from their source of release. They have been detected in water supplies, including drinking water, soil and sediment, and wildlife, where they can bioaccumulate and biomagnify up the food chain [9,16,17,18,19,20]. PFASs in environmental media could well be adsorbed on microplastics also [21]. Humans may be mainly exposed to PFASs through water and diet; ingestion of house dust, inhalation of indoor air, and dermal absorption are other routes of exposure that can contribute significantly, especially in the context of occupational exposure and in contaminated areas, but considerable variations were observed among individuals [22,23,24,25,26,27,28,29,30,31,32].

PFASs have been linked to various adverse human health outcomes even though research findings are sometimes conflicting. Studies have shown associations between PFAS exposure and health problems especially in vulnerable populations, including endocrine disruption, increased risk of some cancers (such as kidney, bladder, thyroid and testicular cancer), cardiac issues, liver metabolic disruption, neurodevelopment effects, immune system impairment, reproductive toxicity, and development effects including altered fetal and postnatal growth [13,33,34,35]. Further investigation into the health impacts of diverse mixtures of PFASs, particularly at low-level, chronic exposure, is essential.

Nowadays, thousands of PFASs continue to be used in numerous categories of application. Among those in which daily human exposure has not yet received much attention in terms of consumer awareness are personal care products and cosmetics. As is well known, some PFASs are commonly added into certain cosmetics and personal care products such as makeup powders, mascaras, anti-aging treatments, lipsticks or foundations, shampoo and hair products, and sunscreen, due to their emulsifying, antistatic, binding, surfactant, stabilizing, water-repellent and long-lasting, film-forming and viscosity-increasing properties [5,36,37]. However, it should be noted that the presence of certain PFASs in cosmetics may in some cases not be intentional due to their presence in raw material impurities or due to the breakdown of intentionally added PFASs that form other types of PFASs [5,38,39,40].

Due to the continuous growth in the utilization of these products, dermal exposure to PFASs could be a significant source of exposure that raises concerns about the associated risk, while ingestion and inhalation appear to play a lesser role. Recent studies suggest that dermal exposure to PFASs through daily contact with consumer products may also play a significant role in exposure, especially to short-chain compounds [32,41,42,43,44,45]. Dermal exposure is a complex process involving the contact and interaction of substances with the skin surface over time. Following skin contact, a substance may penetrate the outermost layer of the skin, permeate the deeper skin layers, or ultimately enter the bloodstream, thereby contributing to systemic exposure [46,47]. The main sources of dermal exposure to PFASs are dust, clothing, textiles and furniture, home products, personal care products and cosmetics [48]. Estimating how much skin exposure contributes to overall human exposure to PFASs is complicated by various factors, the main one being the wide variety of PFASs found in industrial products and the differences in their chemical and physical properties [44]. In addition, it is very likely that many unknown or unquantifiable PFASs are present in common consumer products [49]. Other factors that can influence dermal exposure pathways are lifestyle, age, and sex. Exposure to PFASs from the use of personal care products affects both men and women although women use more personal care products than men, so they are exposed to more unique ingredients daily [41]. Finally, the specific health impacts and exposure levels can vary by sex. An example of age influence is that dermal uptake of PFASs can also be observed in infants by using diapers [50]. Because of these difficulties in assessment and the limited number of studies [44,48], human exposure to PFASs via cosmetics is not yet well understood, as well as the potential effects on human health.

This review aims to assess the current picture of human exposure to PFASs resulting from the use of cosmetics and personal care products by collecting and evaluating the data available worldwide; it intends to highlight critical information gaps, providing a starting point for future research. Several aspects were taken into account, starting with PFAS occurrence in cosmetics and personal care products, dermal absorption of these compounds, human biomonitoring studies on exposure to PFASs, health relevance, and current international regulations.

In this paper, we considered several PFASs, some of which are listed in Table 1.

## 2. Materials and Methods

A thorough literature search was conducted in the PubMed, Google Scholar, ACS Publications, and Science-Direct databases to prepare a narrative review. The search results were updated in May 2026. The search terms included the following: PFAS, human exposure, cosmetics, skin care products, personal care products, and dermal exposure/dermal absorption. Two or three keywords were combined for each search. Existing PFAS use references were also consulted. We conducted further research to contextualize the topic as effectively as possible, highlighting the information gathered and identifying any gaps. Inclusion criteria were: (1) papers published in the last 15 years in scientific journals; (2) studies on external and internal human exposure to PFASs via multiple pathways; (3) papers on the environmental fate of PFASs; (4) studies investigating the presence of PFASs in cosmetics and PCPs using qualitative and quantitative analytical techniques; (5) studies on the potential adverse health effects associated with dermal exposure to PFASs from cosmetics and PCPs; (6) studies on the mechanisms of PFAS absorption through the skin; (7) papers about human biomonitoring studies assessing oral, dermal, or inhalational exposure to PFASs originating from cosmetics and personal care products; and (8) European and non-European regulations currently in force concerning the presence of PFASs in cosmetics and PCPs.

An initial review revealed that ninety studies were suitable for our purposes; most of them are listed in the ‘References’ section. The literature search was limited to articles written in English. The literature published between 2011 and 2026 was regarded; ~70% of the included literature was published in the last three years. Several dozen manuscripts were screened and assessed for eligibility. Additionally, to ensure inclusivity and comprehensiveness, the search incorporated manuscripts that addressed human exposure to PFASs from the use of cosmetics and personal care products and from the environment, diet, and water also. Studies on the chemical composition of personal care products and the resulting skin exposure account for the largest number. Articles concerning bioaccessibility, dermal bioavailability of cosmetics and personal care products, and metabolism of PFASs of interest were regarded as highly significant. All papers underwent an initial filtering process based on their titles, authors and year of publication to avoid duplicates; subsequently, the abstract and a quick read-through of the entire manuscript were decisive in the final decision to include it in this work. Studies deemed irrelevant or, conversely, too specific and detailed for our investigation were excluded. Due to the extensive literature available, we focused on the areas aligned with our specific expertise and ongoing research activities. Consequently, we prioritized publications assessing both the qualitative and quantitative presence of PFASs in cosmetics and personal care products. This approach aimed to identify the most commonly targeted compounds, the relevant analytical techniques, and the levels typically found. To this end, fifteen studies were selected, summarized, and are described in Table 2. No quantitative meta-analysis was conducted in this review paper. Based on these data, we aimed to investigate whether human exposure to PFASs from cosmetics and PCPs is significant within the context of total human exposure, taking into account the contact and absorption mechanisms described in the literature. To achieve a more accurate and relevant assessment, selected ad hoc biomonitoring studies were integrated into the analysis as described below.

## 3. Results and Discussion

As previously mentioned, fifteen studies were selected and are shown in Table 2 in descending order by sampling date: country of sampling, kind of cosmetics/PCPs and number of samples, analytes, and concentrations (if available) are reported.

Although limited in number, the studies vary considerably, starting with the type of fluorinated compound and the number of analytes. Some studies analyze cosmetics to identify those PFASs that are classified as ingredients based on information available to the Food and Drug Administration (FDA), such as perfluoroethyl triethoxysilane, polytetrafluoroethylene (PTFE), tetradecyl aminobutyroylvalylaminobutyric urea trifluoroacetate, trifluoropropyl cyclohexylsiloxane, and trifluoropropyl cyclopentasiloxane. Others, on the other hand, focus on PFASs known and studied for longer, such as PFOA, perfluorobutanoic acid (PFBA), perfluoropentanoic acid (PFPeA), perfluorohexanoic acid (PFHxA), and perfluoroheptanoic acid (PFHpA), because they were found in various types of products and at high concentrations. Emerging PFASs were also analyzed in some products [51]. Additionally, several studies have measured the total fluorine content to identify both per- and polyfluoroalkyl substances (PFASs) and unknown precursors to perfluoroalkyl acids (PFAAs) and perfluorocarboxylic acids (PFCAs) [37,51,52,53,54,55]. The most frequently analyzed cosmetics are sunscreens, foundations and mascara, whereas personal care products (PCPs) include cream, lotion, and wash-free masks, hand cream and body lotion, cleansers, toothpastes, and haircare products. The concentration range of PFASs varies substantially depending on the specific cosmetic product evaluated and the analytical methodology employed. In [56], fifty-nine reusable feminine hygiene product samples from South and North America, Europe and Asia were analyzed; PFASs were detected in 100% of the extracted products, with Σ_42_PFAS in the range 21.4–2200 ng/g. The most abundant compounds were 6:2 and 8:2 fluorotelomer alcohols. A total of 64 beauty products were analyzed in a Chinese study [51]. Thirteen of the 64 products were classified as F-listed, as they contain at least one fluorinated compound in the list of ingredients unlike F-unlisted products, which do not contain fluorinated compounds. Thirty-three PFASs were included in the analysis; PFOA was detected in 30 products, showing the highest detection frequency of about 47% followed by perfluorononanoic acid (PFNA). PFOA and PFNA concentrations were low (except for one product), and no statistically significant difference was found between the two groups (F-listed and F-unlisted). Differences in levels were observed between the two groups for shorter-chain PFASs (C4-C7). The average concentration in the F-listed group was 2.60 μg/g versus 0.281 ng/g in F-unlisted products.

In another study [52], six cosmetics were collected from Swedish stores in 2020. Products were selected based on high levels of total fluorine and PFASs in the list of ingredients. Polar and non-polar PFASs were quantified. Concentrations of residual perfluoroalkyl carboxylic acids (PFCAs) ranged from 0.02 to 29 mg/g, while volatile listed ingredients in three products were measured at levels 876–1323 µg/g. The dermal bioaccessibility assay was also performed.

The presence of PFASs in cosmetics and personal care products has also been documented through the analysis of 21 cosmetic samples from various national and international brands available on the Thai market [57]. PFOS was generally found at low levels (range 1.12–12.07 ng/g); by contrast, PFBS and PFBA had high levels because short-chain PFASs (C4) had replaced long-chain (C8). Among different cosmetic categories, powder products had the highest total PFAS concentration (706.75 ng/g), followed by foundation (186.46 ng/g), blush on (30.69 ng/g) and lipstick (25.66 ng/g) products. Very low levels were found in a European qualitive study in which 11 different PFASs were detected in 765 products, all having PFAS-free formulas [58]. The most frequent compounds were PTFE and perfluorodecalin.

In another study, 57 cosmetic samples purchased from local markets in Guangzhou, China, were analyzed for 17 PFASs determined using ultrahigh-performance liquid chromatography–tandem mass spectrometry (UHPLC-MS/MS) [59]. The concentration range was 0–10.8 ng/g of product, which was lower than that reported by [37] (0–118.1 ng/g) and much lower than that reported by [60] (0–19,000 ng/g). The medium-chain (C7–C10) PFASs were detected with the highest frequency, especially PFOA as in USA products [37].

Short-chain perfluorinated carboxylic acids were the dominant compounds in 45 cosmetics samples purchased on an online shopping platform in China and analyzed for 27 PFASs [61]. Products for pregnant women were included. PFASs were found in all samples; concentrations were in the range 4.05–94.9 ng/g. PFBA and PFPeA were the most frequent PFASs in cosmetics and in general, short-chain PFASs were abundant across all products. These compounds are commonly used to replace longer-chain PFASs due to current regulations [64]. Although they appear to be less bioaccumulative, they are nonetheless highly persistent and very mobile in water. The presence of these compounds in cosmetics is therefore a cause for concern not only because of human exposure, but also because of their potential release into the environment even if they are lower than emissions from other sources [54,61]. The diversity of targeted analytes underlines the absence of standardized reference analytes for assessing contamination levels in mass market products globally. In addition, substantial concentration variability hinders meaningful product-to-product comparison.

An attempt is being made to overcome the lack of target analytes by measuring high total fluorine concentrations as observed in several studies. This is particularly important for those compounds found in high quantities such as 6:2 and 8:2 FTOHs, which are precursors to PFCAs [54]. Analyses of cosmetic ingredient labels confirm that the presence of fluorinated compounds is frequently omitted from packaging, complicating accurate assessments of their widespread use. Consequently, estimating consumer contact levels becomes highly challenging, substantially hindering the assessment of human exposure associated with specific product usage. Without mandatory labeling regulations, consumers are unable to make informed purchasing decisions to mitigate potential PFAS exposure [54]. In Europe, exposure to PFASs from cosmetics is lower than that from other sources, but it could nevertheless be of significant concern, as the concentrations found in some products sometimes exceed the EU limits for PFOA (25 ng/g) and for PFOA-related substances (1000 ng/g) [65]. In summary, PFASs are still detected in some cosmetics. Although overall exposure levels are lower than from other environmental sources, their presence remains a potential health concern due to occasional threshold exceedances and persistent bioaccumulation. Precursors to PFCAs that are known to be harmful were detected in 231 cosmetic products purchased in the U.S. and Canada that were tested for total fluorine [37]. Foundations, mascaras, and lip products contained the highest levels of PFASs, ranging from 0.23 to 10,500 ng/g product weight, with an average and a median of 99 and 591 ng/g product weights, respectively. The 6:2 and 8:2 fluorotelomer compounds were the most commonly detected. In the majority of products, the presence of fluorinated compounds was not disclosed by the ingredient lists. This discrepancy—both in terms of the analytes being analyzed and the concentrations detected—suggests that regulatory measures at a global level are incomplete, remaining fragmented and highly variable from one country to another.

Thirty-nine PFASs and total fluorine were detected in thirty-one CPs from five product categories (cream, foundation, pencil, powder and shaving foam). The most frequent compounds were perfluoroheptanoic acid (PFHpA) and perfluorohexanoic acid (PFHxA) and polyfluoroalkyl phosphate esters (PAPs). Total PAP concentrations (14 compounds) up to 470 µg/g were measured in products listing mixtures of PAPs as an ingredient [55]. PAPs were also detected in a Japanese study together with PFCA compounds in 24 personal care products. The maximum total PFCAs was up to 5.9 µg/g for cosmetics and 19 µg/g for sunscreens [60]. The 6:2 diPAP was quantified in real samples at concentrations ranging from 1.26 to 3.48 ng/g in four of the nine mascaras tested in [62].

The complexity of the formulations of cosmetic and personal care products, the mixture of compounds they contain, combined with the diversity in the number and structure of fluorinated compounds, make it very difficult to assess human exposure to this class of substances. Research on PFASs in cosmetics and personal care products is limited, characterized by small sample sizes, a focus on specific product categories, and a lack of analytical methods and comprehensive toxicological data. The studies presented indicate a shortage of consistency in the results, a wide variation in the fluorinated substances analyzed, the lack of officially standardized analytical methods, and widely differing concentration levels both across product types and within the same product category. In particular, defining specific analytes and standardizing analytical methods would allow for direct comparisons between PFAS levels in cosmetics and PCPs. This current limitation precludes a precise assessment of dermal and inhalational exposure, despite a clear understanding of the underlying absorption mechanisms. Fluorine mass balance calculations currently appear to be the best approach for estimating the quantity of fluorinated compounds that are precursors to both identified and unidentified PFASs [55].

To assess human exposure to PFASs, it is essential to understand the role of cosmetics in internal exposure. The crucial question is whether PFASs in cosmetic products can cross the human skin barrier and what the fractional absorption is [45]. Research on dermal exposure of per- and polyfluoroalkyl substances in personal care products is limited [43], and the dermal absorption fraction is uncertain. However, some in vivo and in vitro studies investigated dermal penetration, permeation and the mechanism of PFAS dermal absorption. Studies are often based on animal or 3-D human skin equivalent models, which do not fully replicate the physiology of real human skin [42,43,66,67]. In [68], the skin permeability of PFASs present in sunscreen products was assessed using mouse skin: PFOA, PFHxA, and PFBA were evaluated using a flow-through diffusion system in ex vivo mouse skin as described by [67]. According to the results, two sunscreen formulations (W/O and O/W types) that most enhanced PFAS dermal absorption were selected for a subsequent experiment. In this 30-day repeated exposure study in mice, these substances were shown to penetrate the skin barrier. In another study [69], again employing an in vitro continuous-flow diffusion technique, skin permeabilities for perfluorooctanoic acid (PFOA), perfluorohexanoic acid (PFHxA), and perfluorobutanoic acid (PFBA), all 14C-labeled, were determined on porcine skin.

Transdermal absorption of PFOA was shown in an in vivo study where ^13^C_4_-PFOA was mixed into sunscreen that was applied on the whole skin of a volunteer. The maximum level in the serum sample was measured 22 days after the application, representing 9.4% of the PFOA level resulting from the volunteer’s background exposure [70]. While these findings by Abraham et al. provide valuable preliminary insights, further studies are required for validation, given the inherent limitations of a single-subject trial.

The results of the selected studies showed that the dermal absorption of PFASs appears to decrease with the increasing carbon chain length of the PFAS: short-chain PFAS, such as PFBA and PFPeA, can cross human skin in vitro far more easily than long-chain PFASs [42]. The chain length alone cannot explain the absorption of PFASs through the skin. The skin’s stratum corneum acts as a formidable, semi-permeable lipid barrier, and how a PFAS molecule navigates this barrier depends on a multi-factorial matrix of its physicochemical properties and the biological state of the skin [66]: molecular weight, functional groups, pH, ionization state, pKa, volatility, penetration time, and skin barrier integrity should be taken into account in order to properly assess and understand the process. This is a critical issue as the potential for toxicity and the mechanism of toxicity are not yet fully understood. For that purpose, there is an urgent need to standardize analytical methods that take into account the chemical properties of PFASs and the physiological properties of human skin. In particular, in vivo studies appear to be essential for confirming—or refuting—the findings already derived from in vitro studies.

**Figure 1:**
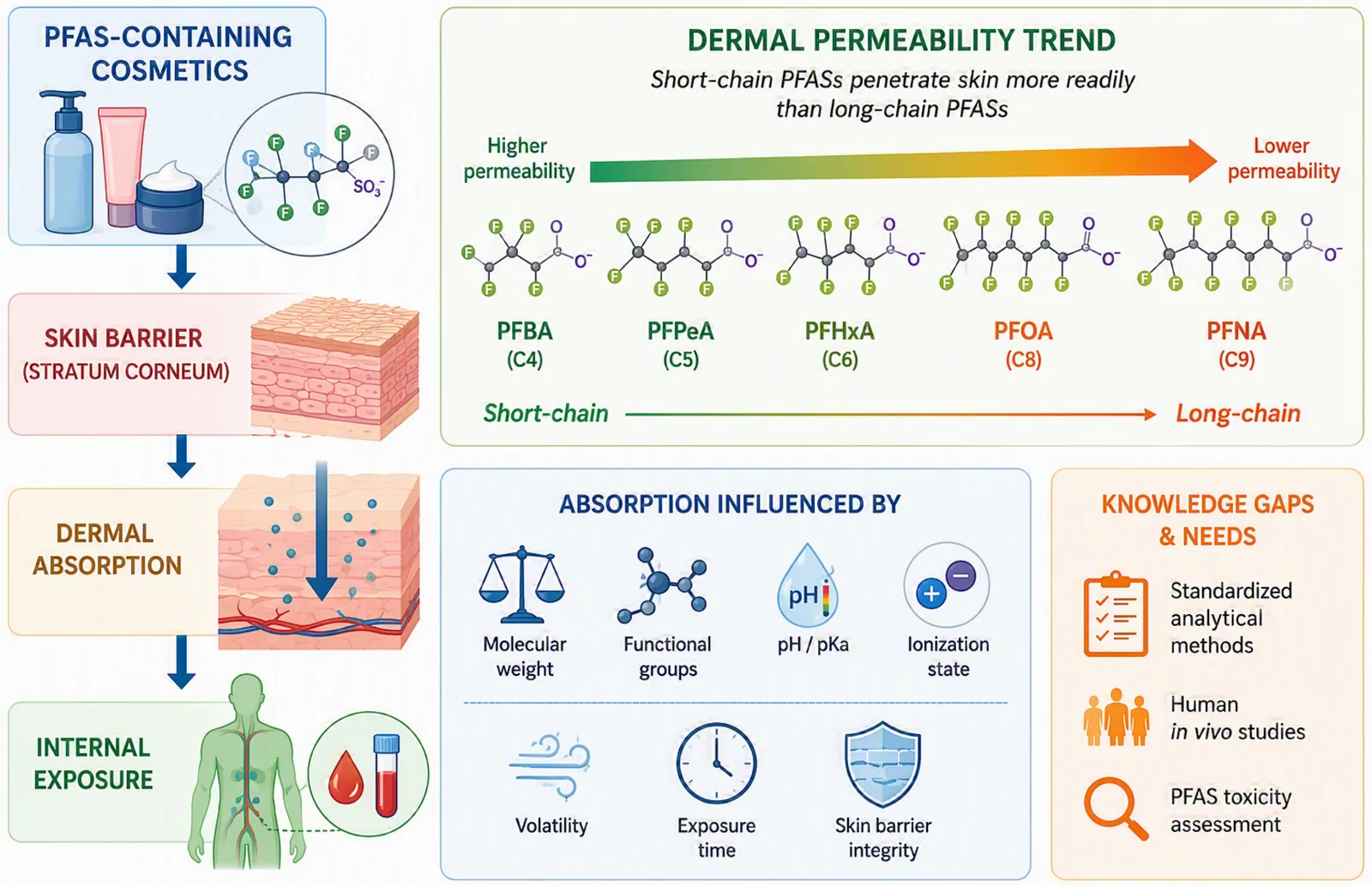
Key steps in the human assessment of dermal exposure resulting from the use of cosmetics and personal care products are illustrated.

To summarize, studies on skin exposure and the associated health risks vary considerably from one another, both in terms of methodology and findings. Figure 1 illustrates the most important steps of the overall assessment process that every study should take into account.

Ad hoc human biomonitoring (HBM) studies have been conducted worldwide to assess exposure to PFASs via oral, dermal, or inhalation routes associated with the use of cosmetics and personal care products. Variations in the specific cosmetics and personal care products tested, as well as the target PFAS evaluated, may contribute to differences in findings. In a study on pregnant African American women, foundation use was significantly associated with the serum levels of PFOA and PFNA. Other key predictors of serum PFAS concentrations identified in this population included parity, educational attainment, tobacco use, home age, and drinking water source [71]. Another study has highlighted the contribution to PFAS exposure arising from the use of personal care products: in [72], the main sources of exposure contributing to serum PFAS concentrations, in particular dietary intake and the daily use of products, were examined in a representative sample of Korean adults. Diet and the use of food contact materials were identified as significant factors contributing to overall PFAS levels in adults. Analyses stratified by sex revealed that shower gel was a primary source of exposure in men, whilst the use of cosmetics was a major source in women, particularly for PFHxS. The most comprehensive study, carried out in Norway on a sample of 44 men and 100 women, aimed to describe PFAS concentrations and evaluate their association with diet and personal care product (PCP) use. Significant differences between males and females were found regarding the frequency and type of PCPs that were used. Positive associations were established between perfluorotridecanoic acid (PFTrDA), 6:2diPAP, PFBS, and PFDS and sunscreen use, and PFTrDA, PFUnDA, and PFHpA and the use of lip gloss by females. Other positive relations were found for PFNA and PFOA in women and for PFOSA in men regarding the use of mouthwash [73]. Similar findings were reported in Spanish donor women, where the sum of PFOA, PFOS, PFNA, and PFHxS concentrations in breast milk samples was positively associated with foundation use. The use of skincare and hair products, cosmetics, perfumes, and deodorants was associated with higher concentrations of both long-chain and short-chain PFASs [74].

In an American maternal–infant study, associations between PCP use and PFAS concentrations in prenatal plasma and human milk were investigated. The study demonstrated that the use of specific personal care products, including hair dyes, was associated with elevated levels of PFASs in plasma during pregnancy and in breast milk post-partum [75]. Future research on the role of PCPs in PFAS exposure should account for specific product types, usage frequency, application timing, and formulation differences. These findings can support PFAS regulatory policy and help individuals lower their chemical exposure.

Although in the general population diet is the most common source of exposure, our findings show that fluorinated ingredients in beauty products may introduce high levels of PFASs and pose a potential exposure risk warranting further attention. It is difficult to draw conclusions on the cutaneous absorption of these molecules as the results of studies differ so widely. Further research is necessary to investigate the long-term impacts of using such cosmetics and personal care products and the associated risks to human health. In particular, toxicokinetic studies that assess the mechanisms of affinity between plasma proteins and PFASs are essential for evaluating the distribution of these analytes in tissues; studies aimed at determining of elimination half-lives of PFASs and renal reabsorption, and the metabolism of these compounds and their precursors in humans, should be carried out. Furthermore, targeted human biomonitoring studies involving the general population, particularly the most vulnerable groups, such as children and pregnant women, and workers, such as beauticians and hairdressers, should be a priority for assessing dermal exposure to PFASs.

## 4. Current Regulations

To date, regulatory restrictions and substitution measures have been implemented with the aim of reducing or eliminating the presence of PFASs in cosmetics and in PCPs. The international regulatory framework is very complex and is changing very rapidly. Several countries, including New Zealand, USA, Canada, the European Union, and UK, have already enacted regulations about the chemical limits in cosmetics. New Zealand has decided that the use of all PFASs (over 10,000 chemicals) in cosmetic products will be completely banned from 1 January 2027. From that date, it will no longer be possible to import or manufacture cosmetics containing these substances intentionally added. Furthermore, the sale and supply of PFAS-containing cosmetics will cease by 31 December 2027, and all existing products must be disposed of by 30 June 2028 [76]. Across the US, several states have imposed strict limits on intentionally added PFASs in cosmetics. In California, Colorado, Minnesota and Washington, the total ban on the sale of cosmetics containing added PFASs came into force on 1 January 2025, whilst New York State implemented a similar ban from the end of 2024. From 1 January 2027, a similar regulation will also become effective in New Hampshire, Oregon and Rhode Island [38]. Information relating to each country is shown in Table 3.

The Canadian Environmental Protection Act (CEPA) [77] is Canada’s chemical regulatory framework with the aim of assessing risks associated with chemical substances, managing their use and protecting both human health and the environment. It plays a direct role in how substances are assessed, managed, and, ultimately, also allowed for use in cosmetic products. From 2027, a total ban on PFASs in cosmetics will come into force; meanwhile, the government has required all Canadian companies to declare the import, manufacture or use of over 312 specific PFASs. Strict penalties are in place for anyone who includes PFASs that are already subject to restrictions on make-up products without prior authorization.

The Regulation (EC) No 1223/2009 [78] is the key piece of European legislation governing the manufacture, marketing and safety of all cosmetic products placed on the market within the European Union. Its purpose is to protect human health and ensure a uniform single market. The regulation, together with the REACH regulatory framework [65] and the directives on Persistent Organic Pollutants [64], prohibits the most widely used and hazardous PFASs (such as PFOA, PFOS, and PFHxS) from being used as ingredients in cosmetics on the European market. Furthermore, the European Chemicals Agency (ECHA) is currently assessing a proposal to ban the entire family of over 10,000 PFASs. In particular, the ECHA’s opinion follows pressure from Sweden, Denmark, Germany, Norway, and the Netherlands to take urgent action to ban PFAS use due to its environmental and health impact. The European Commission will decide on whether to act upon the ECHA’s opinion by the end of 2026. Furthermore, the regulation requires manufacturers to use permanent, clearly legible and visible labels. Ingredients, including PFASs, must be listed in descending order of weight according to the INCI (International Nomenclature of Cosmetic Ingredients) system.

Unlike the European Union, which is moving towards a complete phase-out of the entire class of PFASs, the UK’s regulation of PFASs in cosmetics is undergoing a highly complex transition phase, adopting a more cautious and fragmented approach. Indeed, while Northern Ireland continues to automatically align with the European Union’s stricter restrictions, in Great Britain, only those compounds already classified as Persistent Organic Pollutants (POPs) under the UK POPs Regulation [79] are strictly prohibited. PFOS, PFOA and PFHxS (and their salts and related compounds) must not be included in cosmetic formulations, either as intentional ingredients or as contaminants. In Table 4, the current regulations are listed.

Despite the differences in the regulations in force across various countries, there is a clear determination to eliminate these compounds from cosmetics and personal care products, as their toxicity raises concerns for both human and environmental health.

## 5. Conclusions and Perspective

Based on the literature review, the limited number of studies, the large amount and structural diversity of PFASs, and the lack of analytical methods and comprehensive toxicological data, it is difficult to comprehensively assess human exposure to this class of contaminants resulting from the use of cosmetics and PCPs. To mitigate this uncertainty, further research is required. Given the breadth of the issue, it would be useful to identify priority areas for research. In our view, identifying the PFASs in a cosmetic product that are of the greatest toxicological concern and occurrence could be an excellent starting point. From there, the development and validation of an analytical method would enable comparisons to be made between different studies. At the same time, research into the key factors influencing dermal absorption is essential. Finally, data on the toxicity resulting from prolonged dermal exposure are indispensable.

Proper product labeling and legislation focused on safeguarding public health ensures transparency, enabling consumers to make highly informed choices.

## Figures and Tables

**Table 1:** Analytes, acronyms, Chemical Abstracts Service (CAS) registry numbers, and structures are reported.

Analytes	Acronym	CAS Number	Structures
***Perfluoroalkylcarboxylic acids***	*PFCAs*		
Perfluorobutanoic acid	PFBA	375-22-4	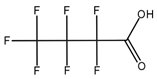
Perfluoropentanoic acid	PFPeA	2706-90-3	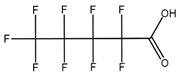
Perfluorohexanoic acid	PFHxA	307-24-4	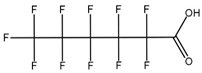
Perfluoroheptanoic acid	PFHpA	375-85-9	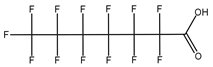
Perfluorooctanoic acid	PFOA	335-67-1	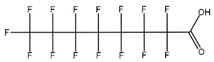
Perfluorononanoic acid	PFNA	375-95-1	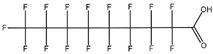
Perfluoro-2-Propoxypropanoic Acid	PFPrOPrA (or HFPO-DA)	13252-13-6	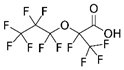
Perfluoroundecanoic acid	PFUndA	2058-94-8	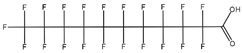
Perfluorotridecanoic acid	PFTrDA	72629-94-8	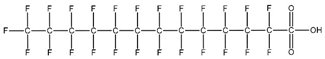
Perfluorotetradecanoic acid	PFTDA	376-06-7	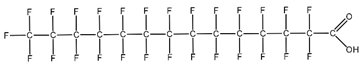
***Perfluoroalkylsulfonic acids***	*PFSAs*		
Perfluorobutanesulfonic acid	PFBS	375-73-5	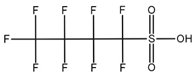
Perfluorohexanesulfonic acid	PFHxS	355-46-4	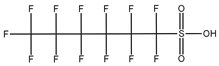
Perfluorooctanesulfonic acid	PFOS	1763-23-1	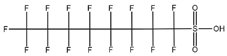
Perfluorodecanesulfonic acid	PFDS	2806-15-7	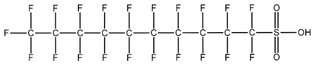
***Other PFASs***			
6:2 chlorinated polyfluoroalkyl ether sulfonate	6:2 Cl-PFAES	73606-19-6	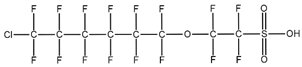
Perfluoroethylcyclohexane sulfonate	PFECHS (or PFEtCHxS)	335-24-0	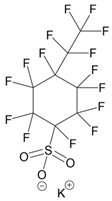
Polytetrafluoroethylene	PTFE	9002-84-0	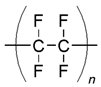
Perfluorodecalin	PFD	306-94-5	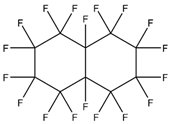
6:2 Fluorotelomer alcohol	6:2 FTOH	647-42-7	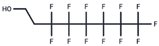
8:2 Fluorotelomer phosphate diester	8:2 diPAP	678-41-1	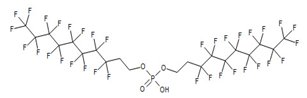
6:2 fluorotelomer phosphate diester	6:2 diPAP	57677-95-9	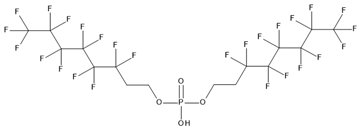
Perfluorooctanesulfonamide	PFOSA	754-91-6	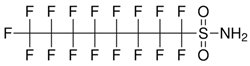
Polyfluoroalkyl phosphate esters	PAP	N/A	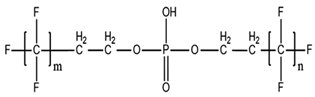

**Table 2:** Selected studies on PFASs in cosmetics and personal care products (PCPs): country of sampling, kind of cosmetics/PCPs and number of samples, analytes and range of concentration (if available) are shown.

N. of Study.	Country of Sampling	Manufacturer	Country of Manufacture	Kind of Cosmetic/PCP and Number of Samples	Analytes and Range of Concentration (If Available)		Analytical Method	Ref.
1	Canada and USA (Indiana and Michigan)	Ulta Beauty, Sephora, Target, Bed Bath & Beyond, and Shoppers Drug Mart	*Data not available*	231 cosmetics	53 PFAS concentrations ranged from 0.23 to 10,500 ng/g product weight, with an average and a median of 99 and 591 ng/g product weights, respectively		PIGE analysis + target analysis (LC/MS)	Whitehead et al. 2021 [37]
2	China	*Data not available*		20 diapers for infants	PFOA (range: <2.34 × 10^−3^–6.458 × 10^−2^ ng/g) 6:2 PAP (range: <1.11 × 10^−3^–8.82 × 10^−3^) FHEA (range: <3.3 × 10^−4^–1.15 × 10^−2^)		Target analysis (LC/MS)	Sun et al. 2024 [50]
3	China	*Data not available*	China, Japan, South Korea, Europe and North America	64 cosmetics and PCPs	33 PFASs: PFOA and PFNA high detection frequency. Σ_target_PFAS ranged from 0.0154 ng/g to 17.1 μg/g (median 9.01 ng/g)		Target analysis (LC/MS) + TOP Assay	Lv et al. 2025 [51]
4	Sweden	L’Oreal Paris, The body shop, Inglot, Peter thomas roth, Glamglow, Living proof	*Data not available*	6 cosmetics	PFCA range 0.02–29 mg/g		Target analysis (LC/MS)	Namazkar et al. 2024 [52]
5	California (USA)	Walmart, Target, iHerb, Sephora, and Amazon	*Data not available*	38,000 cosmetics	2–34 target PFASs		Qualitative study	Balan et al. 2024 [53]
6	Sweden	Table S2 (taken from Putz et al. 2022 [54])		45 cosmetics	Total fluorine (TF), extractable organofluorine (EOF), and 21 target PFAS		Qualitative study	Putz et al. 2022 [54]
7	Sweden	Table S3 (taken from Schultes et al. 2018 [55])	*Data not available*	31 cosmetics	39 PFASs	Σ_14_PAP max value: 470 µg/g	Organic fluorine and total fluorine by combustion ion chromatography (CIC) + target analysis (LC/MS)	Schultes et al. 2018 [55]
8	South and North America, Europe and Asia	Table S1 (taken from Wicks et al. 2025 [56])		59 reusable feminine hygiene products	Σ_42_PFAS: 21.4–2200 ng/g Highest value for 6:2 FTOH: 1380 ng/g Lowest value for PFTDA: 1.12 ng/g		Total fluorine screening via Particle-Induced Gamma-Ray Emission (PIGE) spectroscopy + target analysis (LC/MS)	Wicks et al. 2025 [56]
9	Thailand	*Data not available*	Italy, Japan, Indonesia, Thailand, Germany, Canada, Taiwan, U.S.A., Belgium	21 cosmetics	The total PFAS concentration ranged from 1.98 to 706.75 ng/g		Pressurized liquid extraction (PLE) for target analysis (LC/MS)	Keawmanee et al. 2024 [57]
10	France	*Data not available*		765 cosmetics	PTFE and perfluorodecalin were the most frequent		Qualitative study	Celine et al. 2024 [58]
11	China	*Data not available*		57 cosmetics	The total concentration of PFASs ranged from 0 to 10.8 ng/g		Target analysis (LC/MS)	Tang et al. 2023 [59]
12	Japan, France, USA, and Korea	*Data not available*	Japan, France, USA, and Korea	24 cosmetics and PCPs	9 PFCAs	Σ_9_PFCA: 140–19,000 ng/g	Target analysis (GC-MS)	Fujii et al. 2013 [60]
13	China	*Data not available*		45 cosmetics	Σ_27_PFAS: 4.05–94.9 ng/g		Multi-wall carbon nanotube (MWCNT) extraction + target analysis (LC/MS)	Lin et al. 2023 [61]
14	USA, Italy, Luxembourg and Russia	*Data not available*	USA, Italy, Luxembourg and Russia	9 mascaras	PFBS, 4:2 FTS, PFHxA, PFHpA, PFOA, PFOS, 8:2 FTS, 6:2 diPAP (1.26 to 3.48 ng/g)		Solid-phase microextraction (SPME) + target analysis (LC/MS)	Olomukoro et al. 2026 [62]
15	Canada	*Data not available*		38 cosmetics	200 PFASs; media values: Σ PFSAs: 7.93 ng/g Σ PFCAs: 111 ng/g		SPE column + target analysis (LC/MS)	Harris et al. 2022 [63]

**Table 3:** US legislation concerning the presence of intentionally added PFASs in cosmetics is shown.

State	Statutory Code/Citation	Enacting Legislation	Effective Date	Link
California	Cal. Health & Safety Code § 108980	Assembly Bill 2771 (2022)	1 January 2025	https://leginfo.legislature.ca.gov/faces/billTextClient.xhtml?bill_id=202120220AB2771 (accessed on 4 September 2026)
Colorado	C.R.S. § 25-15-604(1)(a)(VI)	House Bill 22-1345	1 January 2025	https://leg.colorado.gov/bills/HB22-1345 (accessed on 3 September 2026)
Minnesota	Minn. Stat. § 116.943 (*Amara’s Law*)	House File 2310 (2023)	1 January 2025	https://www.revisor.mn.gov/statutes/cite/116.943 (accessed on 4 September 2026)
Washington	Wash. Rev. Code (RCW) § 70A.560.020	House Bill 1047 (2023) (Toxic-Free Cosmetics Act)	1 January 2025	https://legiscan.com/WA/text/HB1047/id/2812309 (accessed on 4 September 2026)
New York State	N.Y. Envtl. Conserv. Law § 37-0117/Title IV	Senate Bill S5648A/Assembly Bill A6292	31 December 2026	https://legiscan.com/NY/bill/S05648/2023 (accessed on 4 September 2026)
New Hampshire	N.H. Rev. Stat. Ann. (RSA) § 149-M:64	House Bill 1649 (2024)	1 January 2027	https://legiscan.com/NH/text/HB1649/id/3003140 (accessed on 4 September 2026)
Oregon	Or. Rev. Stat. (ORS) § 431A.855	Senate Bill 546 (2023) (Toxic-Free Cosmetics Act)	1 January 2027	https://legiscan.com/OR/bill/SB546/2023 (accessed on 4 September 2026)
Rhode Island	R.I. Gen. Laws § 23-18.18-3	Senate Bill 2152/House Bill 7356 (2024)	1 January 2027	https://legiscan.com/RI/text/H7356/id/3008263 (accessed on 4 September 2026)

**Table 4:** The table lists current legislation in non-US countries. Dates of bans are also specified (if available).

Country	Date of Ban	Reference and E-Address of Regulatory Claims
New Zealand	1 January 2027	[76]; https://www.epa.govt.nz/hazardous-substances/rules-notices-and-how-to-comply/specific-substance-guidance/cosmetics/updated-rules-for-cosmetics/ (accessed on 4 September 2026)
Canada	1 January 2027	[77]; https://www.canada.ca/en/environment-climate-change/services/environmental-enforcement/acts-regulations/purpose.html (accessed on 4 September 2026)
Europe	Not defined	[78]; https://eur-lex.europa.eu/legal-content/IT/TXT/?uri=CELEX%3A32009R1223 (accessed on 4 September 2026)
United Kingdom	Not defined	[79]; https://www.gov.uk/guidance/using-persistent-organic-pollutants-pops (accessed on 4 September 2026)

## Data Availability

No new data were created or analyzed in this study. Data sharing is not applicable to this article.

## Supplementary Materials

The following supporting information can be downloaded at: https://www.mdpi.com/article/10.3390/toxics14100930/s1, Table S1: List of feminine hygiene brands tested and region of purchase [56]; Table S2: Overview of cosmetic products purchased from different stores in Stockholm (Sweden) in September 2020 for analysis in the present study [54]; Table S3: List of samples, brand and product names and fluorinated ingredient (INCI nomenclature) [55].
