# Supplementary material for: Per- and Polyfluoroalkyl Substances in Cosmetics and Personal Care Products: State of the Art in Human Exposure
Source: Toxics. 2026 Oct 20;14(10):930. doi: 10.3390/toxics14100930 (PMC13709738; doi:10.3390/toxics14100930)
Supplement: Supplementary file 1 [file toxics-14-00930-s001.zip › toxics-4576171-supplementary.pdf]

Table S1. List of feminine hygiene brands tested and region of purchase*

Brands Tested	Region	Brands Tested	Region
Aisle	N. America	Liz	S. America
Always	Europe	M&S	Europe
Always	N. America	MeLuna	N. America
Amazon Basics	N. America	Modibodi	Asia/Pacific
Bali	N. America	Modibodi	N. America
Bambody	Asia/Pacific	Monoprix	Europe
Bambody	N. America	Nett	Europe
Blooming	Europe	No Boundaries	N. America
Body Form	Europe	O.B.	N. America
Carefree	Europe	Pantys	S. America
Cariona	N. America	Poise	N. America
Chantelle	Europe	Rael	N. America
Charlie Banana	N. America	Rejeanne	Europe
Cora	N. America	Renner	S. America
Dear Kate	N. America	Rovtop	N. America
Depend	N. America	Ruby	N. America
DIM	Europe	Schiesser	Europe
DivaCup	N. America	Selenacare	Europe
DRC	Europe	Seventh Generation	N. America
Equate	N. America	Sloggi	Europe
Essity	Europe	Smoon	Europe
Etam	Europe	Speax	N. America
Fleurity	S. America	Stayfree	N. America
Flux	Europe	Tampax	N. America
Goat Union	N. America	TENA	N. America
Herself	S. America	The Honest Company	N. America
Hope	S. America	Thinx	N. America
Inciclo	S. America	Trendix	N. America
Intimus	S. America	Uniqlo	Asia/Pacific
Jockey	N. America	Uniqlo	N. America
Knix	N. America	Vania	Europe
Korui	S. America	Wuka	Europe
Kotex	N. America	Yuper	S. America
Lena	N. America	Zorbies	N. America
Lil-Lets	Europe	—	—

***Ref. [56]**: Wicks, A.; Brady, S.; Whitehead, H.D.; Hedman, T.; Zachritz, A.; Venier, M.; Peaslee G.F. Per- and Polyfluoroalkyl Substances in Reusable Feminine Hygiene Products. *Environmental Science & Technology Letters* **2025,** *12* (8), 924-92. Doi: 10.1021/acs.estlett.5c00553.

Table S2. Overview of cosmetic products purchased from different stores in Stockholm (Sweden) in September 2020 for analysis in the present study.*

Sample name (Sub Category)	Product name	Brand/Company	Fluorinated Ingredient (PFAS INCI)
**Decorative cosmetics**			
Blush/Bronzer/ Contour 1	Hot MAMA! Shadow/Blush	The Balm cosmetics	PTFE
Blush/Bronzer/ Contour 2	CINDY-LOU MANIZER AKA "The Con-tour Artist"	The Balm cosmetics	PTFE
Blush/Bronzer/ Contour 3	FREEDOM SYSTEM Face Blush 25	INGLOT	POLYPERFLUOROMETHYLISOPROPYL ETHER
Blush/Bronzer/ Contour 4	Lasting Finish Soft Color Blush	RIMMEL LONDON	PTFE
Concealer 1	CC C'est Magic anti-redness skin enhancer	L'Oréal Paris	PERFLUOROOCTYL TRIETHOXYSILANE
Concealer 2	CC+ Color Correcting Full Coverage Cream+ Hydrating and Anti-Aging Conclear, Neutral Tan	IT Cosmetics	PERFLUORODECALIN, PERFLUOROHEXANE, PERFLUOROMETHYLCYCLOPENTANE
Eyeliner pen 2	Crayon Yeux Impact Couleur Tenue 16H Color Eye Pencil 4	Yves Saint Laurent	PERFLUORONONYL DIMETHICONE
Eye shadow 1	Eyeshadow Fard à paupières	URBAN DECAY	PTFE
Eye shadow 2	Eye Color Bar	IsaDora	PTFE
Eye shadow 3	Smoke balm	The Balm cosmetics	PTFE
Eye shadow 4	Color sensational	Maybelline New York	PTFE
Eye shadow 5	FREEDOM SYSTEM Eye shadow Matte NF 298	INGLOT	POLYFLUOROMETHYLISOPROPYL ETHER
Eyeliner liquid/gel	AMC Eyeliner Gel 94	INGLOT	POLYFLUOROMETHYLISOPROPYL ETHER

Foundation/BB Cream 1	BB C'est Magic BB cream 5 in 1 skin perfecter	L'Oréal Paris	PERFLUOROOCTYL TRIETHOXYSILANE
Foundation/BB Cream 2	Flawless Satin Foundation SPF 20	SENSAI	TRIFLUOROPROPYL DIMETHICONOL
Foundation/BB Cream 3	C Super balanced makeup	Clinique	C9-15 FLUOROALCOHOL PHOSPHATE
Foundation/BB Cream 4	Fresh Nude Foundation SPF 15, Sahara Light 030	THE BODY SHOP	AMMONIUM C6-16 PERFLUOROALKYLETHYL PHOSPHATE
Lip liner pen 1	Crayon levres 01	CLARINS PARIS	PERFLUORONONYL DIMETHICONE
Lip liner pen 2	Mood Crayon Anger	LINDA HALLBERG	POLYETHYLENE PERFLUORONONYL DIMETHICONE
Loose powder	Loose powder	INGLOT	POLYPERFLUOROMETHY LISOPROPYL ETHER
Mascara	Chubby Lash Fattening Mascara	CLINIQUE	PTFE
Pressed powder 1	FREEDOM SYSTEM Pressed Powder round 30	INGLOT	POLYPERFLUOROMETHY LISOPROPYL ETHER
Pressed powder 2	Smooth Finish Foundation Powder	Laura Mercier	POLYPERFLUOROETHOX YMETHOXYDIFLUOROET HYL PEG PHOSPHATE
**Hair care**			
Hair spray 1	Blowout Styling & Finishing spray	Living proof.	OCTAFLUOROPENTYL METHACRYLATE (OFPMA)
Hair spray 2	Superfine Hair Spray fixatifix	ORIBE	HYDROFLUOROCARBON 152a
Shampoo	Frizz Shampoo	Living proof.	OCTAFLUOROPENTYL METHACRYLATE (OFPMA)

Styling cream	Balm d'Or Heat Styling Shield	ORIBE	C4-18 PERFLUORPALKYLETHYL THIOHYDROXYPROPYLTR IMONIUMCHLORIDE
Treatment 1	Prime Style Extender	Living proof	OCTAFLUOROPENTYL METHACRYLATE (OFPMA)
**Skin care**			
After shave	Ultra Comfort Moisturizing Balm Soothing After Shave	Biotherm Homme	POLYPERFLUOROMETHY LISOPROPYL ETHER
Anti-age cream 1	Revitalift Night Cream	L'Oréal Paris	ACETYL TRIFLUOROMETHYLPHE NYL VALYLGLYCINE
Anti-age cream 2	Snake serum O2	Rodial	PERFLUOROHEXANE, PERFLUOROPERHYDROP HENANTHRENE, PERFLUORODECALIN
Anti-age cream 3	Age Fitness Advanced Toning Anti-aging care	Biotherm Homme	POLYPERFLUOROMETHY LISOPROPYL ETHER
Exfoliator	Scrub & Mask	FILORGA	PERFLUOROHEXANE, PERFLUORODECALIN, PERFLUOROMETHYLCYC LOPENTANE
Eye moisturiser 1	Advanced Génifique Yeux	LANCOME	PTFE
Eye moisturiser 2	Pep-start Eye Cream	CLINIQUE	TRIFLUOROACETYL TRIPEPTIDE-2
Facial moisturiser	Facial Fuel Energizing Moisture Treatment For Men	Kiehl's	POLYPERFLUOROMETHY LISOPROPYL ETHER
Mask 1	Instamud 60-Second Pore refining treatment	GLAMGLOW	ETHYL PERFLUOROBUTYL ETHER, ETHYL PERFLUOROISOBUTYL ETHER

Mask 2	Water Drench Hyaluronic Micro-Bubbling Cloud Mask	PETER THOMAS ROTH	METHYL PERFLUOROBUTYL ETHER, METHYL PERFLUOROISOBUTYL ETHER, PERFLUOROHEXANE, PERFLUOROPERHYDROPHENANTHRENE, PERFLUORODECALIN, PERFLUORODIMETHYLCYCLOHEXANE
Mask 3	Bubblesheet Oxygenating Deep Cleanse Mask	GLAMGLOW	METHYL PERFLUOROBUTYL ETHER
Moisturiser/Face cream 1	REVITALIFT Day Cream SPF 30	L'Oréal	ACETYL TRIFLUOROMETHYLPHENYL VALYLGLYCINE
Moisturiser/Face cream 2	Ultra Facial Oil-free Lotion	Kiehl's	POLYPERFLUOROMETHYLISOPROPYL ETHER
Serum and treatment 1	Beauty Lift Illuminating V-Shaping Serum	LUMENE	TRIFLUOROACETYL TRIPEPTIDE-2
Serum and treatment 2	Blue therapy red algae uplift	Biotherm	ACETYL TRIFLUOROMETHYLPHENYL VALYLGLYCINE
Blank samples (no PFAS on ingredient list)			
Eyeliner pen 1	Color Essence Eye Pencil (celestial)	H&M	-
Treatment 2	Perfect Hair Day	Living proof.	-

***Ref. [54]**: Pütz, K.W.; Namazkar, S.; Plassmannb, M.; Benskin, J.P. Are cosmetics a significant source of PFAS in Europe? product inventories, chemical characterization and emission estimates. *Environ. Sci.: Processes Impacts* **2022**, *24*, 1697-1707. Doi: 10.1039/D2EM00123C.

Table S3. List of samples, brand and product names and fluorinated ingredient (INCI nomenclature)*

Sample ID[a]	Brand	Product name	Fluorinated ingredient
CRE01	L'Oréal	Skinperfection, Correcting Day Moisturiser	PTFE
CRE02	Biotherm	Aquasource, rich cream, dry skin	Polyperfluoromethylisopropyl ether
CRE03	Biotherm	Homme, Aquapower, Oligo-thermal comfort care	Polyperfluoromethylisopropyl ether
CRE04	Biotherm	Skin-best, Cream SPF 15, Normal/Combination Skin	PTFE
CRE05	Garnier	The Miracle Cream, SPF 20, all skin types	PTFE
CRE06	Lumene	Beauty lift, illuminating V-shaping serum	Trifluoroacetyl tripeptide-2
CRE07[b]	Biotherm	Aquasource nutrition, rich balm, very dry skin	—
FOUN01	The Body Shop	Shade adjusting drops darkening	Ammonium C6-16 perfluoroalkylethyl phosphate
FOUN02	Lumene	Nude perfection fluid foundation, 2 Soft Honey, normal to oily skin	C9-15 fluoroalcohol phosphate
FOUN03	The Body Shop	Fresh Nude Foundation SPF 15, 020 Bali Vanilla	Ammonium C6-16 perfluoroalkylethyl phosphate
FOUN04	IsaDora	Hydralight, water-based matte make-up, 57 fair beige	Polyperfluoroethoxymethoxy difluoroethyl PEG phosphate
FOUN05	Lumene	BLUR Foundation longwear, 6 Golden light, all skin types, SPF 15	Perfluorooctyl triethoxysilane
FOUN06	Sensai	Fluid finish lasting velvet, SPF 15	Trifluoropropyl dimethiconol
FOUN07[b]	Lumene	Invisible illumination, instant glow beauty serum, all skin types	—
FOUN08[b]	IsaDora	Wake up make-up, SPF 20	—
FOUN09[b]	The Body Shop	Moisture foundation SPF 15	—
PEN01	H&M	Color Essence Eye Pencil (celestial)	Perfluorononyl dimethicone

POW01	H&M	Eylure Brow Palette, brow trio	PTFE
POW02	IsaDora	Eye color bar	PTFE
POW03	IsaDora	Anti-Shine Mattifying Powder	Polyperfluoroethoxymethoxy difluoroethyl PEG phosphate
POW04	H&M	Face Palette (4 colors)	Polytetfum (PTFE), polytef (PTFE)
POW05[b]	IsaDora	Bronzing powder	—
POW06	Lumene	Luminous matt	Perfluorooctyl triethoxysilane
POW07[b]	H&M	Blusher highlighter palette	—
POW08	IsaDora	Ultra Cover compact powder 6in1	Polyperfluoroethoxymethoxy difluoroethyl PEG phosphate
POW09	Lumene	Longwear blur	Perfluorooctyl triethoxysilane
POW10	H&M	Highlight Palette (4 colors)	Polytetfum (PTFE), polytef (PTFE)
POW11	H&M	Face Palette (3 colors)	Polytef (PTFE)
POW12	Lumene	CC color correcting powder 6 in 1	Perfluorooctyl triethoxysilane
SHAV01	Gillette	Satin Care, Pure & Delicate	PTFE
SHAV02[b]	Gillette	Satin Care, Olay, Violet Swirl Shave gel	—

*__Ref. [55]__: Schultes, L.; Vestergren, R.; Volkova, K.; Westberg, E.; Jacobson, T.; Benskin, J. P. Per- and polyfluoroalkyl substances and fluorine mass balance in cosmetic products from the Swedish market: implications for environmental emissions and human exposure. *Environ. Sci.: Processes Impacts*, **2018**, *20*, 1680. Doi: 10.1039/c8em00368h.

[a] CRE = cream; FOUN = foundation; PEN = pencil; POW = powder; SHAV = shaving foam.
[b] No fluorinated ingredient listed.
